# Should Mesh Plug Use Be Discontinued in Hernia Repair Practices?

**DOI:** 10.7759/cureus.74204

**Published:** 2024-11-22

**Authors:** Mena Louis, Nathaniel Grabill, Jerrell Fang, Oluwasemilore Okunlola, James Chambers

**Affiliations:** 1 General Surgery, Northeast Georgia Medical Center Gainesville, Gainesville, USA; 2 General Surgery, Northeast Georgia Medical Center Braselton, Braselton, USA

**Keywords:** case series, hernia repair, mesh migration, mesh plug complications, surgical outcomes

## Abstract

Mesh plugs are commonly used in inguinal hernia repair due to their perceived efficacy in reducing recurrence rates. However, their use has been associated with significant complications, including mesh migration, chronic pain, infection, hernia recurrence, adhesions, and erosion into adjacent organs. This case series presents three patients who experienced complications from mesh plug migration post-hernia repair. The patients, aged 63, 82, and 90, presented with symptoms ranging from chronic pain and groin bulging to acute-onset pain and recurrent hernias. Diagnostic imaging revealed migrated mesh plugs adhered to critical structures such as the spermatic cord and small bowel. The surgical intervention involved robotic-assisted laparoscopic techniques to excise the migrated mesh plugs and place the new mesh in the preperitoneal space. Postoperative outcomes were stable. A review of the literature supports our findings, emphasizing the multifactorial mechanisms behind mesh migration and its severe clinical implications. Given these risks, we recommend generally avoiding the use of mesh plugs in hernia repair, if possible. Instead, other mesh alternatives and improved fixation techniques should be considered to enhance patient outcomes and reduce the incidence of these complications.

## Introduction

Inguinal hernia repair is one of the most commonly performed surgical procedures worldwide, with millions of operations conducted annually [1]. The introduction of synthetic meshes has significantly reduced recurrence rates, making mesh-based repairs the standard of care [2]. Among various mesh-based techniques, the use of mesh plugs has been widely adopted due to their simplicity and perceived efficacy in providing a robust repair [3].

However, despite their initial popularity, mesh plugs have been associated with a range of complications that can severely impact patient outcomes [4]. Mesh plug migration, chronic pain, infection, hernia recurrence, adhesions, and erosion into adjacent organs are among the most concerning issues reported in the literature [5]. These complications not only lead to significant morbidity but often necessitate further surgical interventions, thereby increasing healthcare costs and patient burden [6,7].

Despite their initial popularity, mesh plugs have been linked to serious complications, including migration, chronic pain, infection, hernia recurrence, adhesions, and erosion into adjacent organs [8]. Mesh migration, in particular, is a serious complication that occurs when the mesh moves from its original placement site to other anatomical regions that is unique to the mesh plug technique compared to other mesh repairs [9]. This can result in organ perforation, chronic pain, and the formation of fistulas, most commonly with the small bowel, colon, and bladder [9,10]. The mechanism behind mesh migration is multifactorial, involving mechanical factors such as improper fixation and inflammatory responses that lead to tissue erosion and migration [11,12].

Chronic pain and inflammation that arise from nerve entrapment, tissue irritation from the mesh material, or chronic inflammatory responses may lead to significantly reduced quality of life and require long-term pain management [13,14].

Mesh infection is another severe complication that can occur immediately post-surgery or as a delayed response due to bacterial colonization [15]. Symptoms typically include fever, localized swelling, redness, and discharge [16]. Management of mesh infections often involves antibiotic therapy, rehospitalization, prolonged hospital stays, and, in severe cases, the removal of the mesh [17].

Hernia recurrence, despite the use of mesh, remains a significant concern. Inadequate mesh fixation, migration, and failure of the surrounding tissue to properly incorporate the mesh contribute to recurrence [18]. Adhesions, or the formation of fibrous bands causing tissues and organs to stick together, are another complication of mesh plugs [19]. These adhesions can cause chronic pain, bowel obstruction, and complications in future abdominal surgeries [20].

Despite these well-documented complications, some surgeons continue to use mesh plugs in hernia repair [21]. This case series aims to highlight the significant complications associated with mesh plugs, present detailed case reports of patients who experienced mesh plug migration, and strongly recommend avoiding their use in hernia repair.

## Case presentation

Case 1

**Figure 1:**
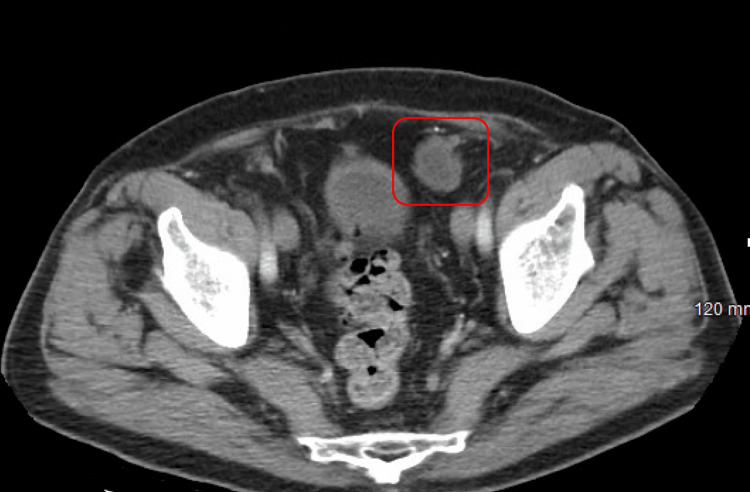
CT of the abdomen and pelvis without contrast (axial view) with recurrent fat-containing left inguinal hernia found in the left anterior pelvis with a 2.7 cm structure consistent with a previous left inguinal hernia repair plug (red box).

**Figure 2:**
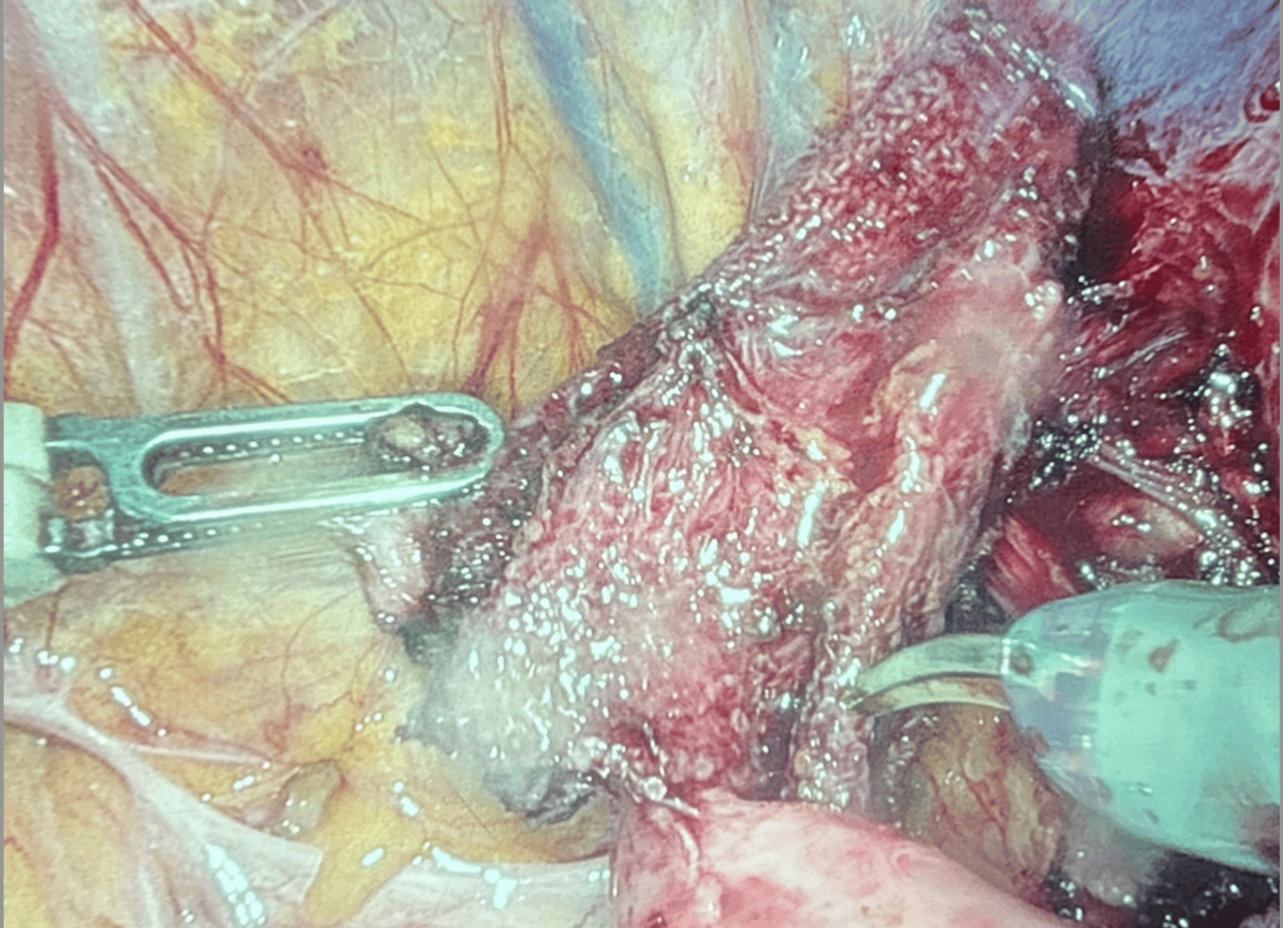
Case 1: intraoperative photo showing a migrated mesh plug found adherent to the cord structures.

An 82-year-old male presented for the evaluation of a recurrent left inguinal hernia. His medical history included type II diabetes mellitus, non-alcoholic liver cirrhosis, hyperlipidemia, and hypertension. Approximately 10 years prior, he had undergone an open left inguinal hernia repair with mesh. Over the last five years, he experienced recurrent hernia symptoms, including frequent bulging in the left groin that fluctuated with physical activity and time of day, along with dull pain and discomfort. Initial laboratory findings were within normal limits (Table 1). A computerized tomography (CT) scan revealed a recurrent fat-containing left inguinal hernia with an approximately 2.7 cm structure consistent with a previously placed mesh plug (Figure 1). During robotic bilateral inguinal hernia repair, a migrated mesh plug was found adhered to the cord structures (Figure 2). The surgical team performed a partial excision of the mesh and placed a new ProGrip mesh. Postoperative recovery was uneventful, and the patient experienced no immediate complications.

**Table 1:** Initial laboratory findings on admission for Case 1.

Test	Result	Normal range
White blood cells	5.9	4.0–11.0 × 10^3^/µL
Hemoglobin	14.8	Men: 13.8–17.2 g/dL; women: 12.1–15.1 g/dL
Platelets	150	150–450 × 10^3^/µL

Case 2

**Figure 3:**
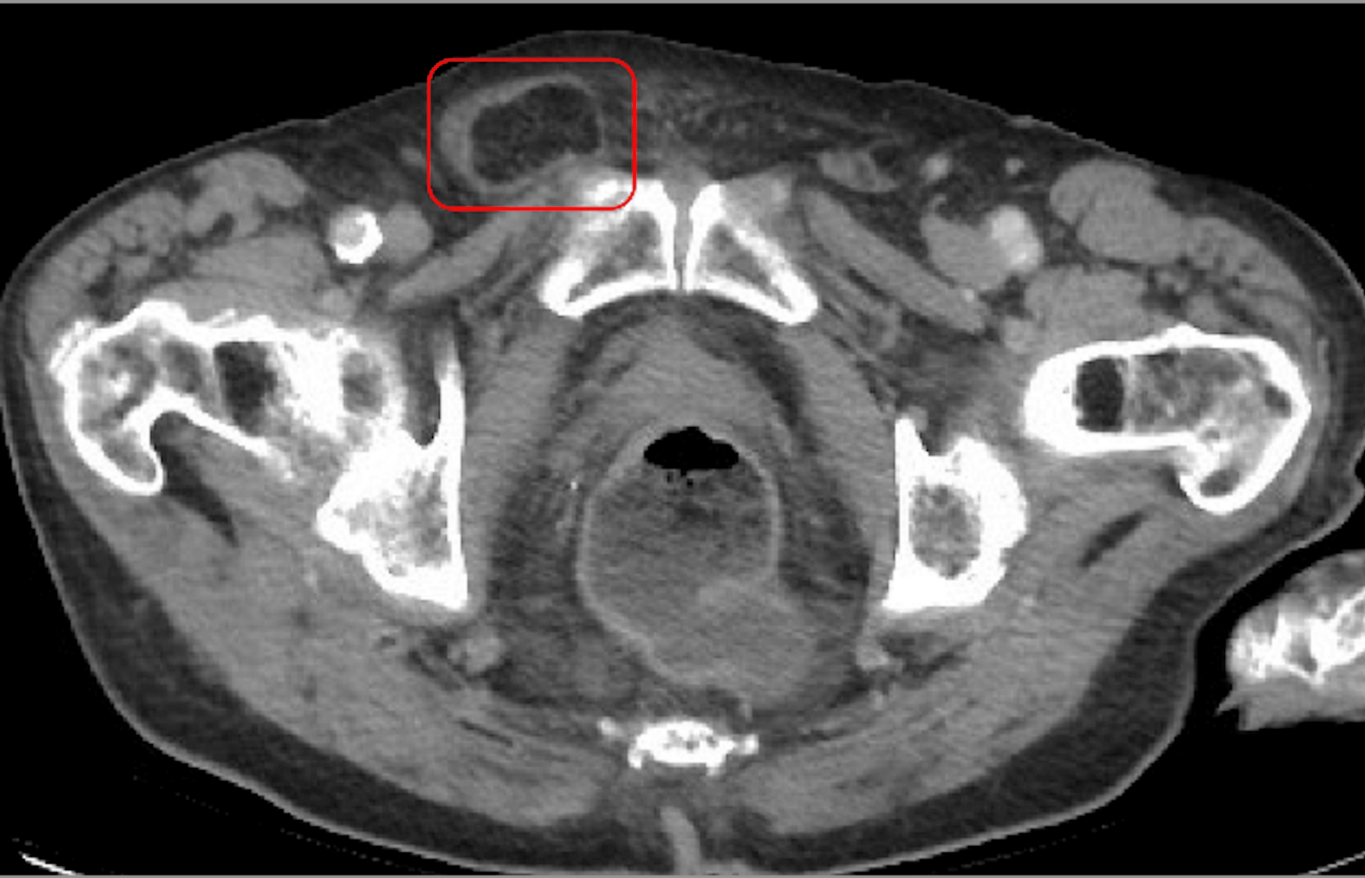
CT of the abdomen and pelvis without contrast (axial view) showing postsurgical changes noted within the left groin related to a previous inguinal hernia repair and a right inguinal hernia identified containing fat and a small volume of fluid (red box).

**Figure 4:**
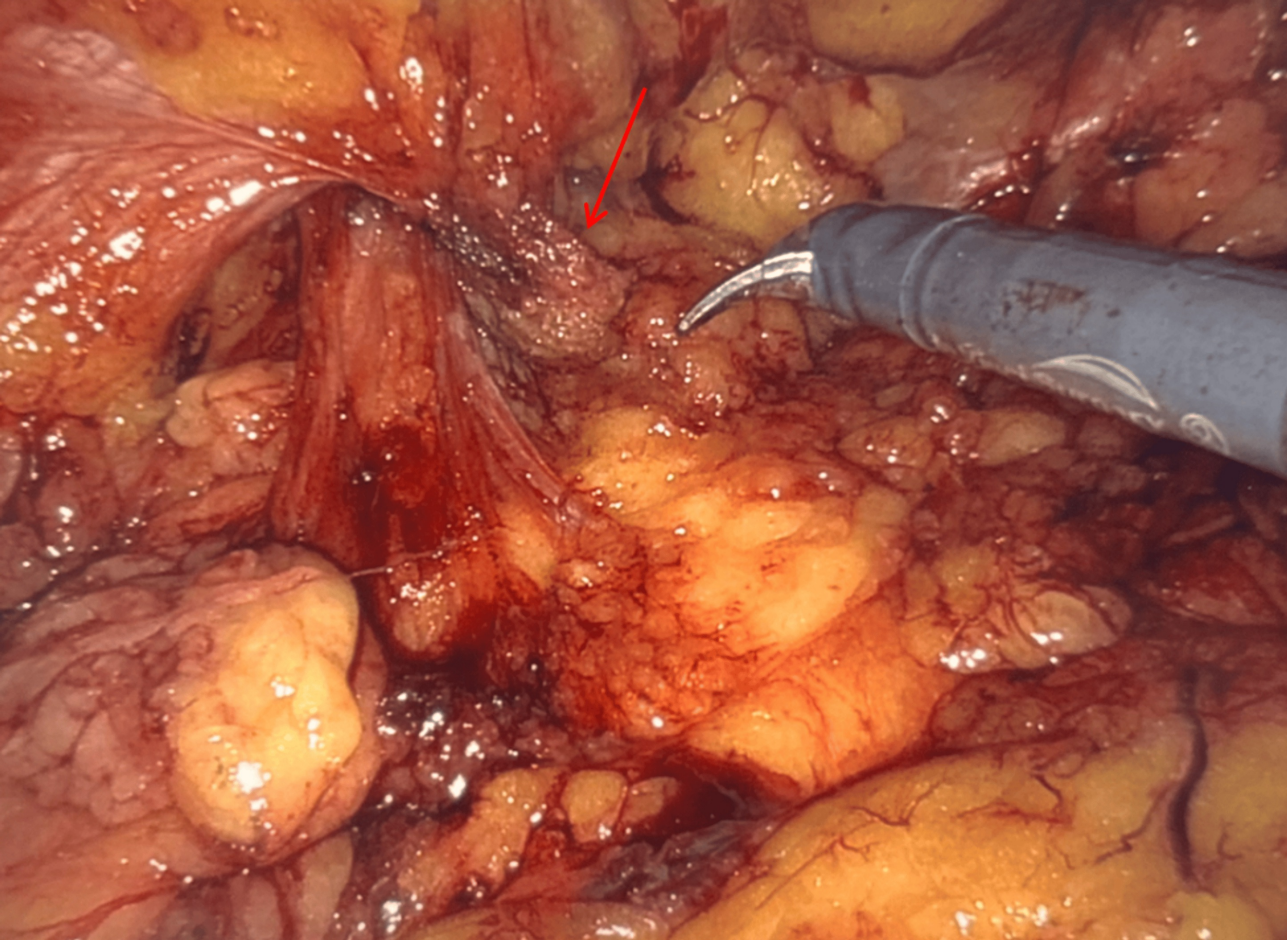
Case 2: intraoperative image of mesh plug (red arrow) found crumpled and adherent to the small bowel.

A 90-year-old male reported a sudden onset of pain in the right groin, radiating across to the left, with a palpable bulge appearing five days prior. His past medical history included congestive heart failure, heart failure with reduced ejection fraction, hypertension, mixed hyperlipidemia, and coronary artery disease. He had previously undergone bilateral inguinal hernia repair and a left hernia repair with mesh over a decade ago. Initial laboratory findings are shown in Table 2. A CT scan revealed a stable right inguinal hernia containing fat and a small volume of fluid, with post-surgical changes in the left groin (Figure 3). During robotic-assisted laparoscopic repair, the previously placed mesh plug was found crumpled and adherent to the small bowel (Figure 4). The crumpled mesh was excised and new ProGrip patches were placed bilaterally. The patient’s postoperative recovery was stable with no immediate complications.

**Table 2:** Initial laboratory findings on admission for Case 2.

Test	Value	Normal range
White blood cells	5.7	4.0–11.0 × 10^3^/µL
Hemoglobin	9.9	Men: 13.8–17.2 g/dL; women: 12.1–15.1 g/dL
Platelets	193	150–450 × 10^3^/µL
pH	7.171	7.35–7.45
pCO_2_	71.0	35–45 mmHg
pO_2_	251	75–100 mmHg
HCO_3_	24.9	22–26 mEq/L

Case 3

**Figure 5:**
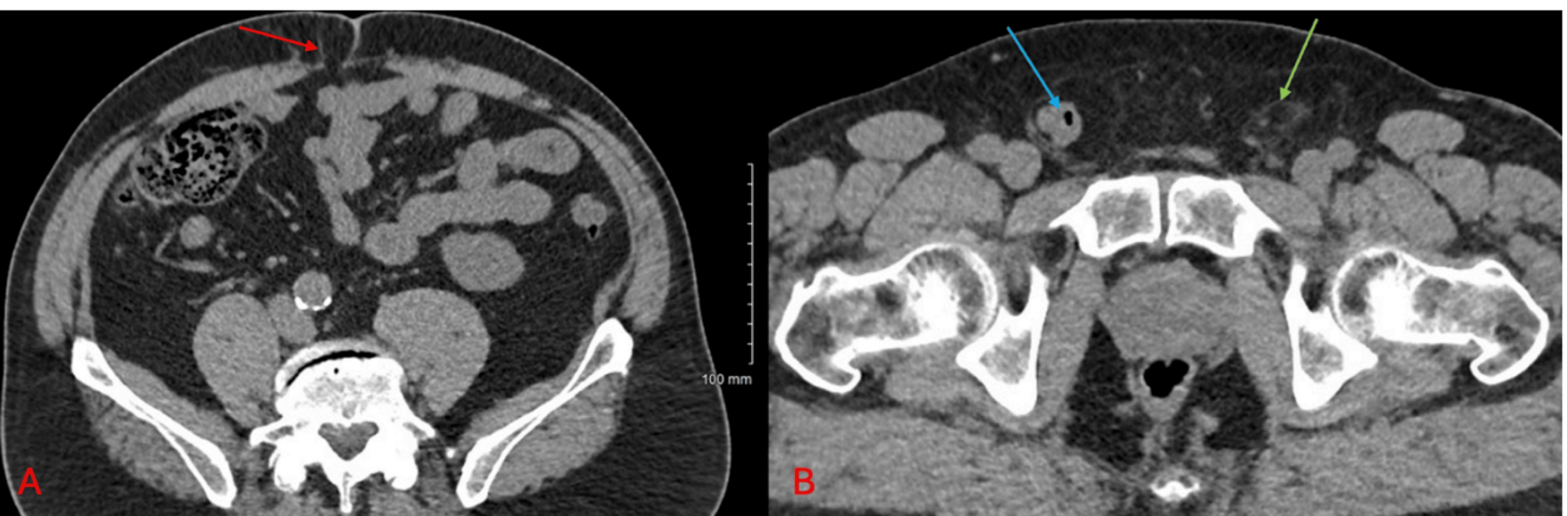
CT of the abdomen and pelvis without contrast (axial view) with a small fat-containing periumbilical hernia in A (red arrow). Panel B shows a small fat-containing left inguinal hernia (green arrow) and a right inguinal hernia containing a loop of the small bowel (blue arrow).

A 63-year-old male presented with a right-sided groin bulge and an umbilical bulge, which had been present for approximately six years and was difficult to reduce. His medical history included hypertension and nephrolithiasis. He had previously undergone umbilical hernia repair and left inguinal hernia repair 24 years ago. Initial laboratory results were normal (Table 3). A CT scan showed a stable small fat-containing periumbilical hernia, a left inguinal hernia, and a small stable right inguinal hernia containing a loop of uncomplicated small bowel (Figure 5). During robotic-assisted laparoscopic bilateral inguinal hernia repair and umbilical hernia repair, a recurrent left inguinal hernia with a synthetic mesh plug adhered near the left internal inguinal ring was discovered. The mesh plug was dissected and new ProGrip meshes were placed bilaterally. The patient had a stable postoperative recovery with no immediate complications.

**Table 3:** Initial laboratory findings on admission for Case 3.

Test	Result	Normal range
White blood cells	6.2	4.0–11.0 × 10^3^/µL
Hemoglobin	15.2	Men: 13.8–17.2 g/dL; women: 12.1–15.1 g/dL
Platelets	237	150–450 × 10^3^/µL

## Discussion

Comparative analysis

Presentation and Initial Findings

Patients in this case series displayed symptoms indicative of complications from mesh plug use, such as pain and bulging. Specifically, the first patient had recurrent symptoms of a left inguinal hernia, including frequent bulging and dull pain over a period of five years. The second patient experienced sudden-onset pain in the right groin with a palpable bulge that subsided after a day but left a persistent lump. The third patient presented with a right-sided groin bulge and an umbilical bulge that had been present for approximately six years and was tender and difficult to reduce. The initial laboratory results were generally normal across the cases, though the second patient had anemia with a hemoglobin level of 9.9 g/dL. Patient comorbidities, including diabetes and hypertension, may influence surgical outcomes by affecting wound healing, increasing infection risk, and contributing to overall complication rates. In this case series, these conditions could have acted as confounding factors, potentially exacerbating the adverse outcomes associated with mesh plug migration.

Diagnostic Approaches

The diagnosis of mesh plug migration and the extent of its complications were primarily determined through clinical examination and imaging studies. For the first patient, a CT scan identified a recurrent fat-containing left inguinal hernia with an approximately 2.7 cm structure consistent with a previously placed mesh plug. In the second case, the CT scan depicted a stable right inguinal hernia containing fat and a small volume of fluid, along with post-surgical changes in the left groin. The third patient’s CT scan displayed a stable small fat-containing periumbilical hernia, a left inguinal hernia, and a small stable right inguinal hernia containing a loop of uncomplicated small bowel.

Surgical Interventions, Findings, and Management

Robotic-assisted laparoscopic surgery, chosen for its precision and minimal invasiveness, was utilized in all cases. The first patient underwent a robotic bilateral inguinal hernia repair where the migrated mesh plug adhered to the cord structures was partially excised and a new ProGrip mesh was placed. The second patient received a robotic-assisted laparoscopic repair of a recurrent right inguinal hernia with mesh, including the excision of a crumpled mesh and repair of a recurrent left femoral hernia with mesh. The third patient underwent a similar procedure for both bilateral inguinal hernia repair with mesh and robotic-assisted laparoscopic umbilical hernia repair without mesh, involving the dissection of a mesh plug adhered near the left internal inguinal ring and the placement of new ProGrip meshes bilaterally.

Postoperative Outcomes

All patients experienced successful recoveries postoperatively, with no immediate complications, which suggests effective surgical management despite the complex nature of mesh plug migration.

Comparative Insights

Surgical consistency was maintained across all cases with the use of robotic-assisted laparoscopic techniques, ensuring precision and minimal invasiveness. The outcomes were uniformly successful, underscoring the significance of meticulous surgical techniques and thorough follow-up care. Despite the common issue of mesh migration, the presentations varied greatly, ranging from gradual symptom development to sudden acute pain. This variation emphasizes the unpredictable nature of complications arising from mesh plug use and supports the recommendation against their use in hernia repair practices.

This case series advocates for a critical reassessment of using mesh plugs in hernia repairs due to the severe complications they can provoke [4]. These complications include mesh migration, chronic pain, infection, hernia recurrence, adhesion formation, and organ erosion, all of which are substantiated through clinical cases supported by extensive literature [6,7].

Mesh plug migration presents complex clinical challenges [20]. This uncommon but severe complication involves mesh moving to critical areas such as the spermatic cord or internal organs, leading to considerable health issues [19]. Often, this requires additional surgical interventions that may involve removing affected organs [22]. Research confirms these findings, showing that mesh migration can lead to severe complications such as organ perforation and the formation of fistulas [23,24].

Chronic pain is another major concern, often arising from nerve entrapment or the irritation caused by the mesh [25]. The cases reviewed document patients enduring considerable discomfort, negatively impacting their quality of life. The inflammatory response to the mesh exacerbates these symptoms, leading to dense adhesions that pose challenges in later surgical procedures [26]. Notably, research suggests that flat mesh repair may be less likely to cause chronic pain compared to mesh plugs, recommending its use over mesh plugs [27].

Mesh infection, while not prominently featured in our series, is a significant risk, ranging from immediate postoperative complications to delayed bacterial colonization that often requires extensive treatment, including antibiotics and potentially mesh removal [15,28]. Hernia recurrence continues to be a significant concern, influenced by factors such as improper mesh placement and integration failures [29]. This emphasizes the need for precise surgical techniques and the careful selection of mesh materials to reduce recurrence risks [30].

Adhesion formation and mesh erosion into adjacent organs add further complexity to postoperative recovery, often requiring complex and multiple surgical repairs [5]. These complications underline the severe consequences of using mesh plugs, especially in open hernia repairs where migration into the abdominal cavity and critical organs is more likely [31].

The HerniaSurge Group’s 2018 international guidelines for groin hernia management recommend the Lichtenstein open mesh repair and laparo-endoscopic techniques such as transabdominal preperitoneal and totally extraperitoneal approaches over the use of mesh plugs. These guidelines advise against the routine use of mesh plugs due to potential complications, including migration and chronic pain [32]. These guidelines favor lightweight mesh to minimize chronic postoperative pain and foreign body sensations without increasing the risk of recurrence [33].

Recommendations

Given the range of severe complications associated with mesh plugs, along with evolving standards of care favoring other techniques and materials, we recommend that the use of mesh plugs in hernia repair be reconsidered and generally avoided. Surgeons should adopt more modern and less problematic alternatives that ensure better outcomes and minimize the risks of severe complications. Continuous patient education and follow-up are essential for the early detection and management of potential complications.

## Conclusions

We strongly recommend avoiding the use of mesh plugs in hernia repair due to the significant risk of complications such as mesh plug migration, chronic pain, infection, recurrence, adhesions, and erosion into adjacent organs. These complications often necessitate further surgical interventions, leading to increased patient morbidity and healthcare costs.

## References

[1] Köckerling F, Simons MP (2018). Current concepts of inguinal hernia repair. Visc Med.

[2] (2002). Repair of groin hernia with synthetic mesh: meta-analysis of randomized controlled trials. Ann Surg.

[3] Fang Z, Ren F, Zhou J, Tian J (2015). Biologic mesh versus synthetic mesh in open inguinal hernia repair: system review and meta-analysis. ANZ J Surg.

[4] Adamonis W, Witkowski P, Smietański M, Bigda J, Sledziński Z (2006). Is there a need for a mesh plug in inguinal hernia repair? Randomized, prospective study of the use of Hertra 1 mesh compared to PerFix Plug. Hernia.

[5] Xie TH, Wang Q, Ha SN (2022). Mesh plug erosion into the small intestine after inguinal hernia repair: a case report. World J Clin Cases.

[6] Moorman ML, Price PD (2004). Migrating mesh plug: complication of a well-established hernia repair technique. Am Surg.

[7] LeBlanc KA (2001). Complications associated with the plug-and-patch method of inguinal herniorrhaphy. Hernia.

[8] Ishikawa S, Kawano T, Karashima R, Arita T, Yagi Y, Hirota M (2015). A case of mesh plug migration into the bladder 5 years after hernia repair. Surg Case Rep.

[9] Haddad A, Yahia DB, Chaker Y, Maghrebi H, Daghfous A, Kacem MJ (2021). Intraperitoneal migrating mesh plug wrongfully taken for right colon cancer: a case report. Int J Surg Case Rep.

[10] Manzini G, Henne-Bruns D, Kremer M (2019). Severe complications after mesh migration following abdominal hernial repair: report of two cases and review of literature. GMS Interdiscip Plast Reconstr Surg DGPW.

[11] Jeans S, Williams GL, Stephenson BM (2007). Migration after open mesh plug inguinal hernioplasty: a review of the literature. Am Surg.

[12] Policarpo F, Rafael AA, Borges MF, Azevedo F (2023). Cecum perforation by plug migration: an unexpected late complication of inguinal hernia mesh repair. J Surg Case Rep.

[13] Ohkura Y, Haruta S, Shinohara H (2015). Laparoscopic plug removal for femoral nerve colic pain after mesh & plug hernioplasty. BMC Surg.

[14] Andresen K, Rosenberg J (2018). Management of chronic pain after hernia repair. J Pain Res.

[15] Shubinets V, Carney MJ, Colen DL (2018). Management of infected mesh after abdominal hernia repair: systematic review and single-institution experience. Ann Plast Surg.

[16] Birolini C, Faro Junior MP, Terhoch CB, de Miranda JS, Tanaka EY, Utiyama EM (2023). Microbiology of chronic mesh infection. Hernia.

[17] Wilson RB, Farooque Y (2022). Risks and prevention of surgical site infection after hernia mesh repair and the predictive utility of ACS-NSQIP. J Gastrointest Surg.

[18] Lowham AS, Filipi CJ, Fitzgibbons RJ Jr, Stoppa R, Wantz GE, Felix EL, Crafton WB (1997). Mechanisms of hernia recurrence after preperitoneal mesh repair. Traditional and laparoscopic. Ann Surg.

[19] Dieter RA Jr (1999). Mesh plug migration into scrotum: a new complication of hernia repair. Int Surg.

[20] Stout CL, Foret A, Christie DB, Mullis E (2007). Small bowel volvulus caused by migrating mesh plug. Am Surg.

[21] Robbins AW, Rutkow IM (1998). Mesh plug repair and groin hernia surgery. Surg Clin North Am.

[22] Sevilla C, Dajani D, Aron M (2017). Migrated mesh plug masquerading as a bladder tumor. J Endourol Case Rep.

[23] Garioud A, Amiot X (2013). Incidental diagnosis of mesh plug migration. Endoscopy.

[24] Scheuer LS, Schnelldorfer T (2015). Inguinal hernia mesh plug migrated into the abdominal cavity. J Gastrointest Surg.

[25] Nienhuijs SW, Boelens OB, Strobbe LJ (2005). Pain after anterior mesh hernia repair. J Am Coll Surg.

[26] Tazaki T, Sasaki M, Kohyama M (2019). Laparoscopic plug removal for chronic pain after inguinal hernia repair using the plug-and-patch technique: a case report. Int J Surg Case Rep.

[27] Donati M, Brancato G, Giglio A, Biondi A, Basile F, Donati A (2013). Incidence of pain after inguinal hernia repair in the elderly. A retrospective historical cohort evaluation of 18-years' experience with a mesh & plug inguinal hernia repair method on about 3000 patients. BMC Surg.

[28] Zou Z, Cao J, Zhu Y, Ma Q, Chen J (2023). Treatment of mesh infection after inguinal hernia repair: 3-year experience with 120 patients. Hernia.

[29] Burcharth J (2014). The epidemiology and risk factors for recurrence after inguinal hernia surgery. Dan Med J.

[30] Tastaldi L, Barros PH, Krpata DM (2020). Hernia recurrence inventory: inguinal hernia recurrence can be accurately assessed using patient-reported outcomes. Hernia.

[31] Na Y, Sun YH, Sun ZC, Xu HM (2017). Mesh erosion into sigmoid colon after inguinal hernia repair. Chin Med J (Engl).

[32] (2018). International guidelines for groin hernia management. Hernia.

[33] Köckerling F, Schug-Pass C (2014). Tailored approach in inguinal hernia repair - decision tree based on the guidelines. Front Surg.

